# Different Effects of Acute and Chronic Hypoxia on Antiviral Capacity of Pulmonary Vascular Endothelial Cells

**DOI:** 10.1155/jimr/1656629

**Published:** 2026-09-03

**Authors:** Robert Szewczyk, Jolanta Kalinowska, Ewelina Sochacka, Maciej Chałubiński, Aleksandra Wardzyńska

**Affiliations:** ^1^ Department of Immunology and Allergy, Chair of Rheumatology, Clinical Immunology and Transplantology, Medical University of Lodz, Lodz, Poland, umed.pl

**Keywords:** antiviral response, asthma, COPD, hypoxia, microvascular endothelial cells

## Abstract

Asthma and chronic obstructive pulmonary disease (COPD) are chronic respiratory diseases associated with inflammation and structural changes in the airways. Hypoxia is a key element of the pulmonary microenvironment, particularly in obstructive diseases, where it can be transient (e.g., during asthma exacerbations) or chronic (COPD). Pulmonary vascular endothelial cells (ECs) are important components of the immune response and (like the epithelium) are exposed to hypoxia and viral infections. Although it has been demonstrated that ECs can be infected by respiratory viruses and that hypoxia can affect their function, the impact of hypoxia on the antiviral capabilities of the endothelium remains poorly understood. This study examined the influence of acute and chronic hypoxia on the antiviral properties of human lung microvascular ECs. Acute and chronic hypoxia upregulated endosomal RNA receptors TLR3 and TLR7, whereas they downregulated cytosolic RIG‐I and MDA5. Acute hypoxia downregulated mRNA and protein expression of IRF3 and IRF7 and inhibited effector antiviral mechanisms, whereas chronic hypoxia induced IRF7, OAS‐1, protein kinase‐R (PKR), and Mx1 at both mRNA and protein levels by flow cytometry. In addition, both hypoxia models caused increased secretion of lymphocyte‐recruiting chemokines, whereas the secretion of cytokines/chemokines responsible for the involvement of monocytes and macrophages was inhibited. Acute hypoxia suppresses antiviral regulatory and effector pathways, whereas chronic hypoxia enhances viral RNA detection and intracellular antiviral mechanisms. These findings highlight the complex role of hypoxia in shaping endothelial immune responses and suggest that, depending on its duration, it may influence susceptibility to viral infections in chronic respiratory diseases.

## 1. Introduction

Asthma and chronic obstructive pulmonary disease (COPD) are the leading causes of morbidity from childhood through old age. The prevalence of asthma ranges from 1% to 29% in the studied population, differing based on location, age, and other factors. COPD affects about 10% of the adult population, is most common among older people, and is the fourth leading cause of death worldwide [1]. Although asthma and COPD differ in terms of their pathogenesis, both diseases are characterized by episodes of tissue hypoxia and are highly susceptible to exacerbations triggered by respiratory viral infections.

In asthma, airway obstruction, bronchoconstriction, and mucus plugging may lead to transient decreases in local oxygen availability. In contrast, COPD is frequently associated with chronic hypoxia resulting from airflow limitation, emphysematous destruction of the alveolar architecture, and impaired gas exchange [2]. Beyond its effects on cellular metabolism, hypoxia contributes to airway and vascular remodeling by promoting endothelial dysfunction, inflammatory signaling, extracellular matrix deposition, and structural alterations of the pulmonary microenvironment. Chronic hypoxia also induces pulmonary vascular remodeling, leading to increased pulmonary vascular resistance and contributing to the development of pulmonary hypertension and, consequently, emphysema, a common complication of advanced chronic lung diseases [3]. Both acute and chronic hypoxia may influence the antiviral capacity of pulmonary vascular endothelium and contribute to viral exacerbations of these diseases.

Hypoxia is a state of an insufficient amount of oxygen to sustain the main functions of organ or tissues. Changes in the availability of oxygen disrupt the normal physiology; hence, oxygen concentration is crucial for maintaining homeostasis and the main functions of organs. Hypoxia is known to be both the triggering and effector factor contributing to both asthma and COPD pathogenesis [4]. Depending on its duration, hypoxia can be classified as acute (typically in the early stages of asthma, without established airway obstruction yet) or chronic (COPD, where these changes are already established). Both have a significant impact on the effects of hypoxia on tissues.

At the cellular level, hypoxia induces molecular mechanisms of transcriptional reprogramming that are dependent on hypoxia‐inducible factors (HIFs). HIF‐1 and HIF‐2 are heterodimers composed of inducible oxygen‐sensitive α subunits and constitutively expressed β subunits. Under normal oxygen pressure, HIF‐α subunits are rapidly posttranslationally hydroxylated by specific oxygen‐dependent prolyl hydroxylases HIF (PHD) [5], recognized by the von Hippel–Lindau tumor suppressor protein (pVHL), and rapidly degraded. Under hypoxic conditions, PHD activity is inhibited, resulting in the accumulation of HIF‐α, which is transported to the cell nucleus and, together with the HIF‐β subunit, forms an active transcription factor (HIF‐1, HIF‐2, or HIF‐3) [6]. The effect of HIF on the vascular system, metabolism, inflammation, and cell apoptosis depends largely on the type of cells and even varies between cells of the same type but different origin. HIF‐1α is considered both a positive and a negative adaptive factor in the context of cell function. Hypoxia is known to induce the release of inflammatory and proangiogenic mediators [7]. Activation of HIF‐1α has been shown to increase the barrier function of epithelial cells [8]. In contrast, hypoxia can damage the blood–brain barrier, leading to increased permeability of brain microvascular endothelial cells (ECs) [9]. Knockdown of HIF‐1α inhibited HIF‐1α‐dependent claudin‐1 expression and epithelial barrier function [10]. On the other hand, HIF‐2α is an essential factor for the maturation of the vascular network [11], and its expression is largely restricted to endothelial and airway epithelial cells. Consequently, HIF‐2α is considered the main regulator of physiological and pathological angiogenic phenotypes that play a key role in COPD [12]. Several studies have shown that stabilization of HIF molecules in a tissue model of hypoxia or oxygen‐independent mechanisms may function as an endogenous adaptive response, balancing inflammation and subsequently restoring normal cellular functions [13].

ECs form the inner layer of blood vessels, act as a semipermeable barrier for immune cells, and maintain homeostasis. In response to viral infections, the endothelium is a powerful source of immune mediators [14], such as interferons, and is involved from the detection to the final self‐eradication of viral infection [15]. Furthermore, ECs may play a significant role in chronic respiratory diseases, such as asthma and COPD. Several studies have suggested the involvement of the endothelium in the pathogenesis of asthma and COPD and their exacerbations [16, 17]. Moreover, endothelial dysfunction has been shown to correlate positively with the severity of COPD.

Pulmonary vascular ECs can be exposed to hypoxia and viral infections. Growing evidence suggests that hypoxia may affect endothelial function [18, 19]. These changes may involve modulation of the cells’ ability to detect and fight against viral infections, which are the most common causes of asthma and COPD exacerbations. Furthermore, hypoxia induces proliferation and vascularization by interacting with the IL‐33 promoter region and producing VEGF‐A, which may play an important role in bronchial wall remodeling.

This study aimed to evaluate the effects of acute and chronic hypoxia on the antiviral properties of human lung microvascular ECs using cell culture methods and models reflecting different durations of hypoxia observed in vivo. This study primarily focused on the impact of various hypoxic conditions corresponding to those observed in asthma and COPD on the elements of the detection and suppression mechanisms as well as the modulation of the antiviral pathways of ECs.

## 2. Materials and Methods

### 2.1. Cell Culture and Induction of Hypoxia

**Figure 1:**
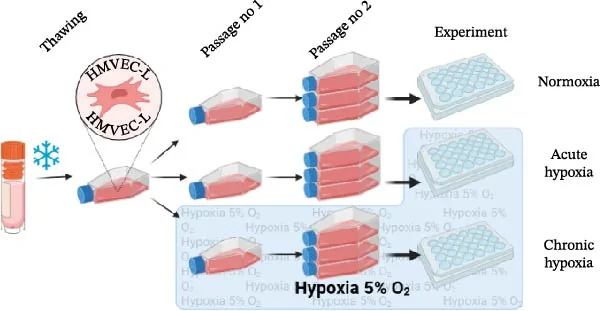
Study design—visualization of cell culture model reflecting three models of oxygen exposure.

Human lung microvascular (ECs) (HMVEC‐L) (CC‐2527, Lonza, Walkersville, MD, USA) were cultured in Endothelial Cell Growth Medium MV 2 (EGM‐MV2) with an included supplement pack (C‐22121, PromoCell GmbH, Heidelberg, Germany). Cells used for each experimental set considering different oxygen availability conditions were proliferated from a single cryovial of liquid nitrogen‐stored HMVEC‐L. After reaching 80%–90% confluence, HMVEC‐L were trypsinized with 0.05% trypsin and 0.02% EDTA (59417C, Sigma Aldrich, St. Louis, MO, USA) and neutralized by Trypsin Neutralizing Solution (CC‐5002, Lonza, Walkersville, MD, USA). Cells seeded on the T‐75 flasks were then divided into three different oxygen availability models: normoxia—cells were for two passages and the experimental period (seeded in a 24‐well plate) cultured under normoxia (21% O_2_, 37°C). The acute hypoxia—cells were cultured for two passages under normoxia (21% O_2_, 37°C) and then, 24 h after seeding into a 24‐well plate, transferred to hypoxia (5% O_2_, 37°C). The chronic hypoxia—cells were for two passages and the experimental period cultured under hypoxia (5% O_2_, 37°C) (Figure 1).

The pre‐experimental period was the same for each model and consisted of two passages, which, depending on the rate of cell growth, lasted between 10 and 12 days. All experimental models began when cells subjected to acute hypoxia were transferred from normoxic to hypoxic conditions (T0). At T0 in each model, the culture medium was replaced to match the specific oxygen availability conditions. To ensure fully hypoxic conditions, all reagents used in the hypoxia chamber were stored under hypoxic conditions for at least 4 h prior to use. For hypoxia studies, a hypoxia chamber (Coy Laboratories, USA) was set to 5% oxygen and maintained at that level with a gas mixture of 5% CO_2_ and balanced nitrogen. All experiments were carried out on cells at the 6–7th passage.

### 2.2. RNA Isolation, Reverse Transcription, and Quantitative Polymerase Chain Reaction (PCR)

Total mRNA was extracted from HMVEC‐L using the ReliaPrep RNA Cell Miniprep System (Z6012, Promega GmbH, Walldorf, Germany) following the manufacturer’s protocol. Subsequently, for cDNA synthesis, the High Capacity cDNA Reverse Transcription Kit (4368813, Thermo Fisher Scientific, Waltham, MA, USA) was used. TaqMan analysis of mRNA expression was performed using the Step One PlusTM real‐time PCR system (Applied Biosystems, Foster City, CA, USA), and the results were normalized to GAPDH (Thermo Fisher Scientific, Waltham, MA, USA) as a housekeeping gene. The following primers of immune checkpoint inhibitors that regulate immune activation and effector functions (PD‐L1, PD‐L2, and LAG‐3), monocyte involvement mediators (MCP‐1), regulators of antiviral response (IRF3 and IRF7), antiviral enzymes (2^′^‐5^′^‐oligoadenylate synthetase 1 [OAS‐1], protein kinase‐R (PKR), myxovirus resistance protein 1 [Mx1]), alarmins (IL‐33 and TSLP), endosomal (TLR3 and TLR7) and cytosolic (RIG‐I, MDA5, and NLRP3) genetic material receptors (Thermo Fisher Scientific, Waltham, MA, USA) were used.

### 2.3. Protein Concentration

Supernatant protein concentrations were measured using Bio‐Plex Pro Human Cytokine Assays (Cat. no. M50‐0KCAF0Y, Bio‐Rad, Hercules, CA, USA) (Table S1) and enzyme‐linked immunosorbent assays (ELISA) following the manufacturer’s instructions: IFN‐β (Human IFN‐beta DuoSet ELISA, Cat. no. DY814, Assay range: –7.8–1000 pg/mL, R&D Systems, Minneapolis, MN, USA), RANTES (Human CCL5/RANTES DuoSet ELISA, Cat. no. DY278, Assay range: 7.8–1000 pg/mL, R&D Systems, Minneapolis, MN, USA), and IL‐8/CXCL8 (Human IL‐8/CXCL8 DuoSet ELISA, Cat. no. DY208, Assay range: –31.3–2000 pg/mL, R&D Systems, Minneapolis, MN, USA).

### 2.4. Analysis of OAS‐1, PKR, and Mx1 by Flow Cytometry

Intracellular OAS‐1, PKR, and Mx1 concentrations were assessed using flow cytometry (BD LSR Fortessa flow cytometer) at 48 and 72 h. HMVEC‐L were fixed and permeabilized using Cytofix/Cytoperm Solution (554722, Franklin Lakes, NJ, USA) for 20 min at 4°C. Subsequently, the cells were washed with Perm/Wash buffer (554723; BD Biosciences, Franklin Lakes, NJ, USA) and incubated with antibodies for OAS‐1, PKR, and Mx1 (sc‐374656, sc‐6282, sc‐271024; Santa Cruz Biotechnology, Dallas, TX, USA) or isotype controls for 30 min at 4°C. After incubation, the cells were analyzed using flow cytometry with BD FACS Diva Software and FlowJo.

### 2.5. Protein Expression by Western Blot

HMVEC‐L cells were lysed using 2 × RIPA buffer (300 mM NaCl, 100 mM Tris‐HCl pH 7.5, 0.2% SDS) combined with 10 × NP‐40 Surfact‐Amps Detergent Solution (Thermo Fisher Scientific), PBS, and 100 × Halt Protease Inhibitor Cocktail (Thermo Fisher Scientific). The samples were then incubated on ice for 30 min, followed by centrifugation (5000 × *g*, 4°C, and 10 min). The total protein concentration in the lysate was quantified using the BCA method (Pierce BCA Protein Assay; Thermo Fisher Scientific). Samples were loaded and separated by sodium dodecyl sulfate‐polyacrylamide gel electrophoresis (SDS‐PAGE) and transferred onto nitrocellulose membranes (Immobilon‐NC, Transfer Membrane, Millipore, Merck). OAS‐1 protein was detected using mouse anti‐OAS‐1 monoclonal antibody (1:1000, sc‐374656, Santa Cruz Biotechnology, USA), PKR with mouse anti‐PKR monoclonal antibody (1:2000, sc‐6282, Santa Cruz Biotechnology, USA), Mx1 with rabbit anti‐Mx1 monoclonal antibody (1:1000, D3W71, Cell Signaling Technology, USA), MDA5 with rabbit anti‐MDA5 monoclonal antibody (1:2000, D74E4, Cell Signaling Technology, USA), RIG‐I with mouse anti‐RIG‐I monoclonal antibody (1:2000, sc‐376845, Santa Cruz Biotechnology, USA), TLR3 with mouse anti‐TLR3 monoclonal antibody (1:2000, sc‐32232, Santa Cruz Biotechnology, USA), TLR7 with rabbit anti‐TLR7 monoclonal antibody (1:2000, D7, Cell Signaling Technology, USA), IRF‐3 with rabbit anti‐IRF‐3 monoclonal antibody (1:2000, D83B9, Cell Signaling Technology, USA), and IRF‐7 with rabbit anti‐IRF‐7 polyclonal antibody (1:2000, 4920, Cell Signaling Technology, USA). Proteins used as loading controls were detected using mouse anti‐β‐actin (1:1000, sc‐47778, Santa Cruz Biotechnology, USA). The secondary antibody used was HRP‐conjugated donkey anti‐rabbit (1:20000, sc‐2313, Santa Cruz Biotechnology, USA) or HRP‐conjugated donkey anti‐mouse (1:20000, sc‐2314, Santa Cruz Biotechnology, USA). The signal was detected by chemiluminescence, and the data were quantified using the ImageJ software. The results were normalized to β‐actin levels (Figure S4).

### 2.6. Statistical Analysis

Due to the small sample size and the inability to assume a normal distribution of the data, the Mann–Whitney *U* test was used to analyze the differences between the two groups. Statistical evaluations were performed using GraphPad Prism software (GraphPad Software, Inc., La Jolla, CA, USA). Differences with *p*  < 0.05 were regarded as significant. In the figures, results are presented as the median ± interquartile range (IQR). Significance between conditions is denoted as  ^∗^, *p*  < 0.05;  ^∗∗^, *p*  < 0.01;  ^∗∗∗^, *p*  < 0.001; and  ^∗∗∗∗^, *p*  < 0.0001.

### 2.7. Artificial Intelligence Generated Content

The manuscript was professionally edited for English language, grammar, and academic tone using Paperpal (Paperpal, Cactus Communications) prior to submission.

## 3. Results

### 3.1. Confirmation of the Effectiveness of the Hypoxia Model

**Figure 2:**
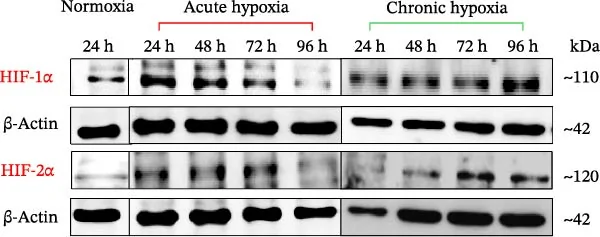
HIF‐1α and HIF‐2α protein expression in HMVEC‐L exposed to acute and chronic hypoxia models.

First, to confirm the effectiveness of hypoxia induction, we determined the expression levels of characteristic proteins that accumulate under hypoxic conditions: HIF‐1α and HIF‐2α. This analysis showed that the HIF‐1α protein accumulated rapidly and reached a maximum level after 24 h in both acute and chronic hypoxia models. HIF‐1α levels decreased notably during prolonged exposure to acute hypoxia and reached lower levels than those observed under normoxic conditions at the last time points. Furthermore, no significant difference was found between HIF‐1α levels in ECs in response to acute and chronic hypoxia. Next, we determined the expression levels of HIF‐2α. HIF‐2α reached its maximum level after 24 and 48 h in the acute hypoxia model and gradually decreased over time (Figure 2). Compared to normoxia, HIF‐2α protein levels were upregulated at every time point of the acute hypoxia experiment (Figure S1). This observation is consistent with the results of previous studies, according to which HIF‐2α is more stable and accumulates longer in vascular ECs under hypoxia. The dynamics of changes in the expression and accumulation of both detected members of the HIF family appear to differ in HMVEC‐L. However, we demonstrated that acute and chronic hypoxia affected the protein expression of HIF‐1α and HIF‐2α.

### 3.2. The Effect of Hypoxia on Receptors Recognizing Viral Genetic Material (Pattern Recognition Receptor [PRR])

**Figure 3:**
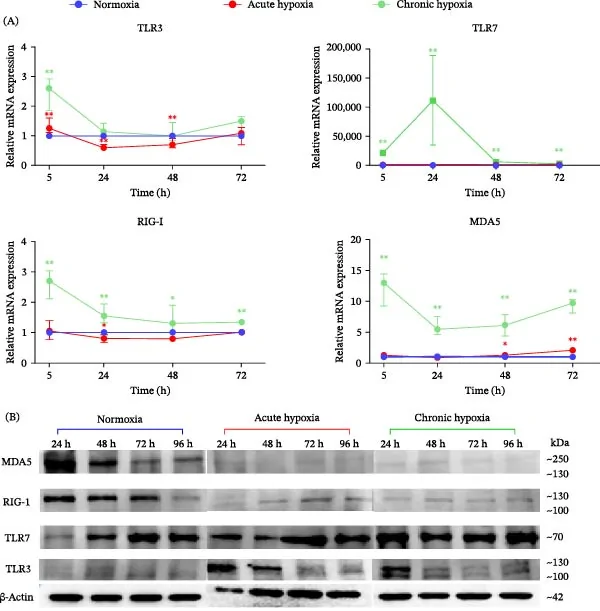
Acute and chronic hypoxia alter endosomal and cytosolic viral RNA receptors. (A) mRNA expression and (B) protein level of endosomal (TLR3 and TLR7) and cytosolic (RIG‐I and MDA5) in ECs under acute and chronic hypoxia. Error bars represent IQR.  ^∗^ *p*  < 0.05 was considered significant. The Mann–Whitney *U* test was used to analyze differences between the groups. Data are representatives of at least three independent experiments as medians ± IQR; *n* = 3–5;  ^∗^ *p* < 0.05,  ^∗∗^ *p* < 0.01, and  ^∗∗∗^ *p* < 0.001.

To determine the impact of hypoxia on the ability of HMVEC‐L to detect viral infections, we assessed the mRNA expression levels and protein levels of intracellular receptors that recognize viral genetic material. As viruses are intracellular pathogens, we focused on the main PRR receptors that recognize double‐stranded and single‐stranded RNA in the two most crucial cellular compartments for viral infection: endosomes (TLR3 and TLR7) and cytosol (MDA5 and RIG‐I). mRNA expression for all selected receptors of the viral genetic material was rapidly induced in all hypoxia models, with the highest level of mRNA expression observed 5 h after the onset of hypoxia, with the exception of TLR7, for which the maximum level was observed after 24 h for ECs under chronic hypoxia (Figure 3A). We observed that ECs increased their expression only at 5 and 72 h in response to acute hypoxia. After a rapid increase at 5 h, their expression gradually decreased between 24 and 48 h, reaching levels lower than those observed in normoxia. In terms of the difference between the effects of short‐term and long‐term hypoxia, chronic hypoxia induced a significantly greater increase in EC mRNA expression of all selected factors and sustained them at elevated levels.

The evaluation of receptors using the western blot method provided new insights into the impact of hypoxia on virus detection by ECs. The results based on protein expression indicated an upward trend in endosomal receptors (TLR3 and TLR7). This observation is consistent with the mRNA expression results. In contrast, the expression of cytosolic receptors (RIG‐I and MDA5) decreased significantly in both acute and chronic models of hypoxia exposure compared to normoxia levels (Figure 3B). Based on these results, we conclude that both hypoxia conditions can affect endothelial receptors for RNA. Acute and chronic hypoxia can increase the ability of ECs to detect viral RNA in the endosomes while inhibiting receptors responsible for the same functions in the cytosol. Interestingly, the same conditions can inhibit the formation of receptors in the cytosol while inducing their gene expression, indicating the effect of hypoxia at the translational state. Taken together, our analysis indicates that acute and chronic hypoxia resulted in the upregulation of endosomal RNA receptors, whereas cytosolic RNA detection mechanisms based on RIG‐I and MDA5 were downregulated.

### 3.3. The Effect of Hypoxia on Regulators and Effector Mechanisms of the Antiviral Signaling

**Figure 4:**
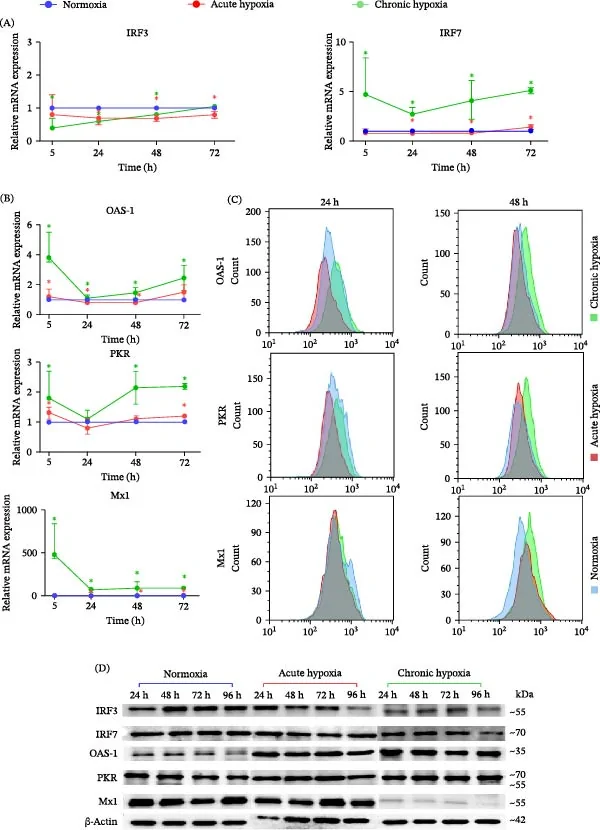
The acute and chronic hypoxia divergently affect regulatory and effector factors of the antiviral signaling of endothelial cells. (A) mRNA expression of IRF3 and IRF7. (B) mRNA expression and (C, D) protein levels (flow cytometry and western blot, respectively) of major effector and regulatory factors of the antiviral phenotype of vascular endothelial cells (ECs) exposed to acute and chronic hypoxia. Error bars represent IQR.  ^∗^ *p*  < 0.05 was considered significant. The Mann–Whitney *U* test was used to analyze differences between the groups. Data are representatives of at least three independent experiments as medians ± IQR; *n* = 3–5;  ^∗^ *p* < 0.05,  ^∗∗^ *p* < 0.01, and  ^∗∗∗^ *p* < 0.001.

PRRs detecting viral genetic material in endosomes and cytosol are considered the most important detectors of viral infection. We demonstrated that acute and chronic hypoxia can affect their expression, which may lead to changes in the regulatory and effector mechanisms of antiviral signaling. To test this hypothesis, we assessed the regulatory and effector factors directly involved in the antiviral properties of ECs. As shown in Figure 4A, acute and chronic hypoxia affected the most crucial IRFs in different ways. Acute hypoxia downregulated both IRF3 and IRF7 mRNA (Figure 4A) and protein (Figure 4D) expression at all tested time points. In the case of IRF3, chronic hypoxia led to a decrease in mRNA levels from 5 to 72 h, whereas IRF7 mRNA was upregulated. Notably, the levels of IRF3 and IRF7 proteins increased in response to chronic hypoxia. These findings support the hypothesis indicating a marked difference between the inhibitory effects of acute and the inducing effects of chronic hypoxia on the antiviral regulatory mechanisms of antiviral activation in ECs. Moreover, our results of further analysis of IRF‐dependent effector mechanisms of the antiviral response, including OAS‐1 and PKR enzymes, are consistent with the observed relationship. We demonstrated that the main enzymes involved in viral RNA degradation (OAS‐1) and viral protein synthesis inhibition (PKR) in response to chronic hypoxia were permanently increased at both the mRNA (Figure 4B) and protein levels (Figure 4C,D). In contrast, acute hypoxia caused a decrease in the levels of all the antiviral enzymes studied. In summary, our analysis indicates that, depending on the duration, hypoxia can divergently affect the regulatory and effector factors of antiviral signaling of ECs, which may directly translate into an actual change in the antiviral potential (Figure S3).

### 3.4. Regulation of the Immune Response Under Hypoxic Conditions

**Figure 5:**
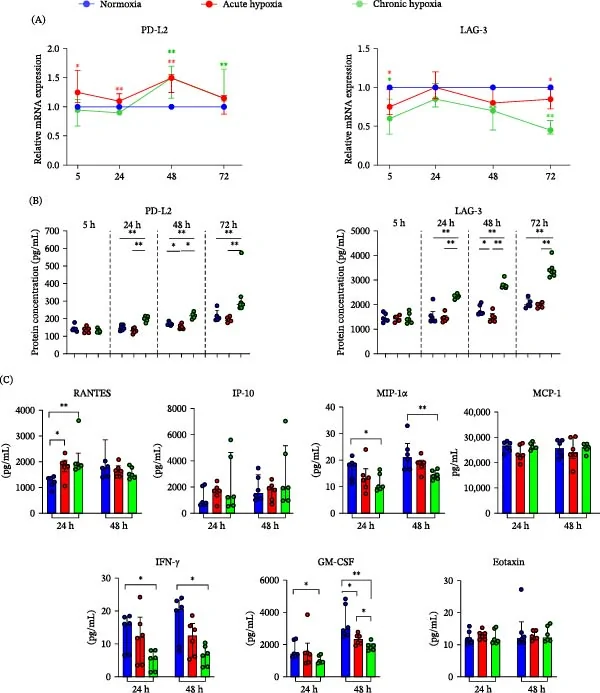
Acute and chronic hypoxia affect crucial antiviral signaling mediators: immune checkpoint inhibitors, cytokines, and chemokines. (A) mRNA and (B) protein release of immune checkpoint inhibitors of ECs under acute and chronic hypoxia models. (C) Protein levels of cytokines and chemokines of ECs under hypoxia conditions. Error bars represent IQR.  ^∗^ *p*  < 0.05 was considered significant. The Mann–Whitney *U* test was used to analyze differences between the groups. Data are representatives of at least three independent experiments as medians ± IQR; *n* = 3–5;  ^∗^ *p* < 0.05,  ^∗∗^ *p* < 0.01, and  ^∗∗∗^ *p* < 0.001.

To fully understand the context of hypoxic conditions and account for inflammatory responses that mediate the antiviral phenotype, we evaluated the expression of checkpoint inhibitors and cytokines/chemokines, which may also influence the abovementioned properties of ECs (Figure S2). PD‐L2 mRNA expression (Figure 5A) and protein (Figure 5B) levels were induced at 24, 48, and 72 h of acute hypoxia, whereas chronic hypoxia caused late upregulation of mRNA (48 and 72 h) and protein (24, 48, and 72 h). In contrast, we did not observe the induction of LAG‐3 mRNA expression in any of the hypoxia models. In contrast to the mRNA results, LAG‐3 protein levels were significantly induced following exposure to chronic hypoxia for 24–72 h. Although we observed significantly elevated protein levels of two immune checkpoint inhibitors regulating immune system responses, we examined cytokines and chemokines mediating possible responses. We selected mediators associated with lymphocyte (RANTES and IP‐10) and monocyte (MIP‐1α, MCP‐1, and GM‐CSF) involvement. Interestingly, as shown in Figure 5C, the release of RANTES protein in the supernatant of both hypoxia‐induced ECs was significantly increased after 24 h. The release of IP‐10 protein also increased after 24 and 48 h in response to acute and chronic hypoxia. In contrast, both exposures to hypoxia caused a decrease in the levels of chemokines associated with the involvement of monocyte‐macrophages, such as MIP‐1α, IFN‐γ, and GM‐CSF. Most importantly, these changes were exacerbated in the chronic hypoxia model. For MCP‐1 and eotaxin, we did not observe any effect of hypoxia on their levels.

## 4. Discussion

In this study, we found that acute and chronic hypoxia differently modulated the antiviral capacity of primary HMVEC‐L. We aimed to evaluate the effects of acute and chronic hypoxia on the antiviral properties of HMVEC‐L using cell culture methods and models reflecting different durations of hypoxia observed in vivo. We focused on the impact of hypoxic conditions corresponding to those most commonly observed in asthma exacerbation (acute hypoxia) and COPD (chronic hypoxia) on the elements of the detection and suppression mechanisms of viral infections. It is difficult to find data on the effect of various types of hypoxic exposure on the antiviral properties of primary pulmonary ECs. Researchers have mainly focused on the effect of hypoxic conditions on cancer cell lines, where hypoxia is the area of greatest interest. Consequently, this is the first study on primary ECs to investigate the modulation of antiviral capacity under conditions of acute and chronic hypoxia.

ECs are increasingly recognized as active participants in immune responses within the lung microenvironment. In addition to their classical role as a vascular barrier, they can produce cytokines, chemokines, and interferons, contributing to antiviral defense and shaping immune cell recruitment. Dysregulation of endothelial immune functions has been implicated in several pulmonary diseases, including viral pneumonia and COVID‐19 [20]. Our previous studies provided important insights into the role of pulmonary vascular ECs in antiviral immunity. Using HMVEC‐L, human rhinovirus HRV16, and human coronavirus HCoV‐229E infection models, we demonstrated that ECs are not merely a passive vascular barrier but actively participate in antiviral defense. Upon viral exposure, ECs recognize viral RNA through pattern‐recognition receptors, such as TLR3, RIG‐I, and MDA5, and subsequently induce interferon‐dependent antiviral signaling, including the effector enzymes OAS‐1 and PKR [15, 21]. In addition, endothelial infection is associated with the release of inflammatory (IL‐1β, IL‐6, and TNF‐α) and lymphocyte‐recruiting chemokines (RANTES, IP‐10, and IL‐8), indicating that these cells may contribute to shaping local immune responses during respiratory viral infections and potentially influence the course of exacerbations in diseases such as asthma and COPD [14, 22, 23].

Our results indicate that acute hypoxia suppresses key regulatory and effector mechanisms of antiviral immunity, whereas chronic hypoxia enhances several antiviral factors, including viral RNA sensing and intracellular antiviral enzyme expression. These findings suggest that hypoxia is not a uniform regulator of endothelial immunity but rather leads to time‐dependent adaptation that may affect responsiveness to viral infections during obstructive lung diseases. Our results are consistent with previous reports, indicating that hypoxia can induce complex immunomodulatory effects depending on exposure duration and cellular context. Chronic activation of HIF signaling has been shown to regulate innate immune responses and may enhance antimicrobial defenses in certain tissues [24]. In ECs, specifically, HIF‐2α has been identified as a key regulator of vascular homeostasis and inflammatory responses under hypoxic conditions [25]. In our study, the accumulation of HIF‐1α and HIF‐2α confirmed the effectiveness of the hypoxia model and suggests that the observed changes in antiviral signaling may be mediated through HIF‐dependent transcriptional regulation.

One of the most important observations made in this study is the divergent regulation of PRRs under hypoxic conditions. Both acute and chronic hypoxia increased the expression of endosomal RNA receptors TLR3 and TLR7, whereas cytosolic RNA sensors RIG‐I and MDA5 were downregulated at the protein level. This indicates that hypoxia may alter the balance between endosomal and cytosolic viral detection mechanisms in ECs. Our previous studies have demonstrated that ECs are capable of sensing viral RNA through both endosomal and cytosolic receptors, leading to the activation of interferon signaling pathways, production of intracellular antiviral mediators, and ultimately, eradication of viral infection by interferon‐dependent mechanisms [15, 21]. However, our findings suggest that hypoxia selectively modifies this system. The observed upregulation of endosomal receptors may represent a compensatory mechanism aimed at maintaining viral recognition when cytosolic sensing pathways are impaired.

Hypoxia‐dependent modulation of PRRs has been described in immune and epithelial cells but not in the primary endothelium. For instance, hypoxia and HIF‐1α signaling have been shown to regulate TLR expression and responsiveness in several cell types [26]. In the respiratory system, hypoxia can enhance inflammatory signaling and innate immune responses by influencing TLR‐mediated pathways [27]. Our results extend these observations by demonstrating that pulmonary ECs respond to hypoxia by selectively enhancing endosomal viral recognition while suppressing cytosolic detection pathways. Such a shift in viral sensing may have functional consequences, particularly during infections with RNA viruses that replicate primarily in the cytoplasm. Interestingly, cytosolic RNA sensors are key components that enable the detection of HRV and RSV infections, which are the most common factors responsible for bronchial asthma exacerbations (60%–70%). This mechanism may be clinically relevant in the context of respiratory RNA viruses, such as rhinoviruses and coronaviruses, whose replication cycles engage cytosolic antiviral sensing pathways. Therefore, hypoxia may differentially affect endothelial responsiveness to viruses depending on their intracellular route of entry, replication strategy, and ability to expose viral RNA to endosomal versus cytosolic sensors.

Another key finding of our study is the divergent regulation of transcription factors IRF3 and IRF7 depending on the duration of hypoxia. Acute hypoxia markedly reduced IRF3 and IRF7 expression, whereas chronic hypoxia increased IRF7 levels and restored antiviral signaling pathways. IRF3 and IRF7 are central regulators of innate antiviral responses and are essential for the induction of antiviral effector genes [28]. Downregulation of IRF3 and IRF7 during acute hypoxia may therefore lead to impaired antiviral defense and increased susceptibility to viral infections. This mechanism may be particularly relevant during acute hypoxic episodes occurring in asthma exacerbations, where viral infections are a major trigger of disease worsening. Our acute hypoxia model was designed to reflect the circumstances occurring during asthma exacerbations when hypoxia is only temporary. Surprisingly, the results showed reduced cytoplasmic sensitivity to viruses and a weakened antiviral capacity of vascular ECs exposed to acute hypoxia. This is consistent with clinical observations indicating that the most common cause of viral exacerbations of bronchial asthma is the aforementioned infections with viruses that replicate in the cytoplasm (e.g. rhinoviruses, RSV).

In contrast, chronic hypoxia enhanced IRF7 expression and induced antiviral effector enzymes, such as OAS‐1, PKR, and Mx1. These proteins play essential roles in the elimination of viral RNA and in the inhibition of viral replication [29], which we proved to be efficient mechanisms for eradicating viral infections in ECs. The induction of these antiviral mechanisms suggests that prolonged hypoxic exposure may lead to adaptive changes that strengthen antiviral immunity in ECs. This adaptive response may be mediated through the long‐term stabilization of HIFs, which regulate numerous genes involved in metabolism, inflammation, and host defense [30]. Therefore, the increased expression of selected antiviral mediators observed under chronic hypoxia, including IRF7, OAS1, PKR, and MX1, may reflect long‐term endothelial adaptation to a hypoxic microenvironment. This distinction is important due to the fact that hypoxia‐adapted ECs may acquire transcriptional and signaling properties that are not equivalent to a simple extension of acute hypoxic exposure. On the other hand, it is well known that hypoxia disrupts other mechanisms, causing immune cells to be reprogrammed and take on a pro‐inflammatory character, which ultimately leads to immune dysfunction.

The above findings may have important clinical implications for obstructive lung diseases. Hypoxia is a common feature of diseases such as asthma and COPD, particularly during exacerbations [31]. However, the temporal pattern and pathophysiological background of hypoxia differ between these conditions. In asthma, hypoxia is usually acute, transient, and often reversible, occurring mainly during exacerbations as a consequence of bronchoconstriction, mucus plugging, ventilation–perfusion mismatch, and virus‐induced airway inflammation. Viral infections are the leading cause of asthma exacerbations, and impaired antiviral responses in airway cells have been widely documented [32]. Therefore, acute hypoxia may reflect the microenvironment experienced by pulmonary ECs during virus‐triggered asthma exacerbations. Our data suggest that acute hypoxia may further weaken the antiviral capacity of ECs, potentially facilitating viral replication in cytosol, endothelial activation, and amplification of airway inflammation during exacerbations.

In contrast, chronic hypoxia is more characteristic of advanced COPD, where persistent airflow limitation, small airway disease, airway remodeling, and impaired gas exchange expose pulmonary vascular and ECs to long‐term oxygen deprivation. COPD is closely related to emphysema, although these terms are not synonymous. Emphysema represents one of the major pathological phenotypes of COPD and is characterized by destruction of alveolar walls and enlargement of distal airspaces, leading to a reduced gas‐exchange surface area and contributing to chronic hypoxia [3]. Thus, the chronic hypoxia model used in our study may be particularly relevant to the endothelial microenvironment observed in COPD, especially in patients with emphysematous changes. In this context, chronic hypoxia may induce adaptive mechanisms that partially restore antiviral activity, possibly reflecting a compensatory response of ECs to prolonged hypoxic stress. Viral exacerbations are also clinically relevant in COPD, where respiratory viruses such as rhinoviruses, RSV, and influenza viruses contribute to acute worsening of symptoms [33]. Thus, in COPD, chronic hypoxia may represent a persistent background condition, whereas respiratory viral infections may act as acute triggers superimposed on an already hypoxia‐adapted pulmonary vascular microenvironment.

Another important aspect of our findings concerns the modulation of cytokine and chemokine secretion. Both acute and chronic hypoxia increased the secretion of chemokines associated with lymphocyte recruitment, particularly RANTES (CCL5) and IP‐10 (CXCL10), while reducing the production of mediators involved in monocyte and macrophage recruitment, such as GM‐CSF and MIP‐1α. These results suggest that hypoxia may alter the composition of immune cell infiltration within the pulmonary microenvironment. Chemokines, such as CXCL10 and CCL5, are critical for the recruitment of T lymphocytes and natural killer cells during viral infections [34]. Therefore, increased secretion of these mediators may promote adaptive antiviral immunity.

In contrast, despite enhanced lymphocyte recruitment, hypoxia induced the expression of immune checkpoint molecules, such as PD‐L2 and LAG‐3, at the protein level. Immune checkpoint pathways regulate immune activation and prevent excessive inflammation; however, they may also contribute to immune suppression during chronic diseases [35]. The simultaneous induction of lymphocyte‐recruiting chemokines and inhibitory checkpoint molecules observed in our study suggests that hypoxia may promote immune cell recruitment while limiting their activation. This dual regulatory mechanism may represent an adaptive strategy for controlling inflammation in chronically hypoxic tissues.

Despite these important findings, some limitations of this study should be taken into consideration. First, the study was conducted using an in vitro model of primary ECs, which does not fully reflect the complexity of the pulmonary microenvironment. Our results can not directly conclude that these endothelial changes functionally reprogram T cells, macrophages, or other immune‐cell subsets. Interactions with epithelial cells, immune cells, and other circulating mediators may significantly influence antiviral responses in vivo. Future studies using coculture models with T cells, macrophages, or other immune populations will be necessary to determine whether the endothelial secretory phenotype observed under hypoxia translates into functional changes in immune‐cell activation, polarization, and antiviral activity. Second, although the study focused on molecular pathways associated with antiviral immunity, functional experiments using viral infection models are necessary to confirm the biological consequences of changes in antiviral signaling induced by hypoxia. Therefore, our findings should be interpreted as hypoxia‐dependent changes in the basal antiviral and inflammatory phenotype of the pulmonary endothelium. This study design allowed us, first, to accurately assess the direct effects of acute and chronic hypoxia and subsequently to evaluate the impact of additional viral stimulation or stimulation via interferon. Conducting such studies is the next part of the project, which aims to first determine the effects of different exposures to hypoxia and then functionally translate them into an active infection model with the most commonly observed clinically relevant viruses. Third, the study analyzed a single oxygen concentration (5% O_2_), whereas oxygen levels in lung tissue can vary significantly depending on the severity of the disease and local blood supply. During preliminary studies using 10% oxygen, we did not observe any significant changes in pulmonary vascular ECs (data not shown). Nevertheless, oxygen availability at 5% O_2_ is a commonly used methodological model for studying the effects of hypoxia on respiratory cells [36, 37]. Furthermore, given the exploratory nature of our study, we used a commercially available panel encompassing a broad range of cytokines, although some mediators were undetectable in our experiments.

In conclusion, our findings demonstrate that hypoxia exerts duration‐dependent effects on the antiviral properties of pulmonary ECs. Acute hypoxia suppresses antiviral regulatory and effector pathways, whereas chronic hypoxia enhances viral RNA detection and intracellular antiviral mechanisms. Our findings highlight the complex role of hypoxia in shaping endothelial immune responses and suggest that time‐dependent oxygen deprivation may influence the antiviral properties of the pulmonary endothelium in chronic respiratory diseases. Future studies should therefore explore the functional consequences of hypoxia‐induced changes in endothelial antiviral pathways using viral infection models. In addition, the investigation of HIF‐dependent signaling pathways and their pharmacological modulation may provide further insights into the mechanisms linking hypoxia and antiviral immunity. Such studies may also identify potential therapeutic targets to improve antiviral responses during exacerbations of obstructive lung diseases.

## Author Contributions

**Robert Szewczyk:** concept, methodology, experiments: RT‐qPCR, ELISA, multiplex, flow cytometry, western blot, data analysis, visualization, manuscript preparation. **Jolanta Kalinowska**: cell culture, data analysis, experiments: RT‐qPCR, western blot. **Ewelina Sochacka**: western blot. **Maciej Chałubiński**: fundraising, preliminary analysis of results. **Aleksandra Wardzyńska**: final analysis, review, supervision.

## Funding

This research was funded by The National Science Centre (Grant UMO‐2021/43/B/NZ5/00973).

## Conflicts of Interest

The authors declare no conflicts of interest.

## Supporting Information

Additional supporting information can be found online in the Supporting Information section.

## Supporting information


**Supporting Information** Figure S1: HIF‐1α and HIF‐2α protein expression in HMVEC‐L exposed to acute and chronic hypoxia models.  ^∗^ *p* < 0.05 was considered significant. The Mann–Whitney *U* test was used to analyze differences between the groups. Data are representative of at least three independent experiments, with medians ± IQR; *n* = 3–5;  ^∗^ *p* < 0.05,  ^∗∗^ *p* < 0.01, and  ^∗∗∗^ *p* < 0.001. Figure S2: Hypoxia exposure affected alarmins, immune response inhibition factor and NLRP3 sensor. mRNA expression of alarmins (IL‐33 and TSLP), NLRP3 and checkpoint inhibitor PD‐L1 of HMVEC‐L exposed to acute and chronic hypoxia. Error bars represent IQR.  ^∗^ *p* < 0.05 was considered significant. The Mann–Whitney *U* test was used to analyze differences between the groups. Data are representatives of at least three independent experiments as medians ± IQR; *n* = 3–5;  ^∗^ *p* < 0.05,  ^∗∗^ *p* < 0.01, and  ^∗∗∗^ *p* < 0.001. Figure S3: Hypoxia results in protein accumulation of chosen factors: OAS‐1, PKR, Mx1, IRF3, IRF7, TLR3, TLR7, RIG‐I, and MDA5. Densitometry analysis of all factors assessed by western blot technique. ECs were exposed to acute and chronic hypoxia for the time periods presented on *x* axis. The changes in protein levels of all assessed factors were evaluated by western blot normalized to β‐actin and total protein levels. Error bars represent IQR.  ^∗^ *p* < 0.05 was considered significant. The Mann–Whitney *U* test was used to analyze differences between the groups. Data are representatives of at least three independent experiments as medians ± IQR; *n* = 3–5;  ^∗^ *p* < 0.05,  ^∗∗^ *p* < 0.01, and  ^∗∗∗^ *p* < 0.001. Figure S4: Western blot uncropped images with loading controls. Figure S5: An example of a full gating strategy of the flow cytometry analysis. Table S1: BioPlex Pro Human Cytokine Assay properties.

## Data Availability

The data that support the findings of this study are available from the corresponding author upon reasonable request.

## References

[1] de Oca M. M. , Perez-Padilla R. , and Celli B. , et al.The Global Burden of COPD: Epidemiology and Effect of Prevention Strategies, The Lancet Respiratory Medicine. (2025) 13, no. 8, 709–724, 10.1016/S2213-2600(24)00339-4. 40684784

[2] Halpin D. M. G. , Masekela R. , and Vogelmeier C. F. , et al.Addressing the Global Challenges of COPD and Asthma: A Shared Vision From the Global Initiative for Chronic Obstructive Pulmonary Disease (GOLD) and the Global Initiative for Asthma (GINA), European Respiratory Journal. (2026) 67, no. 2, 10.1183/13993003.02244-2025, 2502244. 41198399

[3] Kaminsky D. A. and Irvin C. G. , The Physiology of Asthma-Chronic Obstructive Pulmonary Disease Overlap, Immunology and Allergy Clinics of North America. (2022) 42, no. 3, 575–589, 10.1016/j.iac.2022.04.001. 35965046

[4] Zhou W. , Yu T. , Hua Y. , Hou Y. , Ding Y. , and Nie H. , Effects of Hypoxia on Respiratory Diseases: Perspective View of Epithelial Ion Transport, American Journal of Physiology-Lung Cellular and Molecular Physiology. (2022) 323, no. 3, L240–L250, 10.1152/ajplung.00065.2022. 35819839

[5] Greer S. N. , Metcalf J. L. , Wang Y. , and Ohh M. , The Updated Biology of Hypoxia-Inducible Factor, EMBO Journal. (2012) 31, no. 11, 2448–2460, 10.1038/emboj.2012.125. 22562152 PMC3365421

[6] Bakleh M. Z. and Al Haj Zen A. , The Distinct Role of HIF-1α and HIF-2α in Hypoxia and Angiogenesis, Cells. (2025) 14, no. 9, 10.3390/cells14090673, 673. 40358197 PMC12071368

[7] Castillo-Rodríguez R. A. , Trejo-Solís C. , Cabrera-Cano A. , Gómez-Manzo S. , and Dávila-Borja V. M. , Hypoxia as a Modulator of Inflammation and Immune Response in Cancer, Cancers. (2022) 14, no. 9, 10.3390/cancers14092291, 2291. 35565420 PMC9099524

[8] McClendon J. , Jansing N. L. , and Redente E. F. , et al.Hypoxia-Inducible Factor 1α Signaling Promotes Repair of the Alveolar Epithelium After Acute Lung Injury, American Journal of Pathology. (2017) 187, no. 8, 1772–1786, 10.1016/j.ajpath.2017.04.012. 28618253 PMC5530913

[9] Fischer S. , Wobben M. , Marti H. H. , Renz D. , and Schaper W. , Hypoxia-Induced Hyperpermeability in Brain Microvessel Endothelial Cells Involves VEGF-Mediated Changes in the Expression of Zonula Occludens-1, Microvascular Research. (2002) 63, no. 1, 70–80, 10.1006/mvre.2001.2367. 11749074

[10] Masterson J. C. , Biette K. A. , and Hammer J. A. , et al.Epithelial HIF-1α/Claudin-1 Axis Regulates Barrier Dysfunction in Eosinophilic Esophagitis, Journal of Clinical Investigation. (2019) 129, no. 8, 3224–3235, 10.1172/JCI126744. 31264974 PMC6668670

[11] Koh M. Y. and Powis G. , Passing the Baton: The HIF Switch, Trends in Biochemical Sciences. (2012) 37, no. 9, 364–372, 10.1016/j.tibs.2012.06.004. 22818162 PMC3433036

[12] Shimoda L. A. and Semenza G. L. , HIF and the Lung: Role of Hypoxia-Inducible Factors in Pulmonary Development and Disease, American Journal of Respiratory and Critical Care Medicine. (2011) 183, no. 2, 152–156, 10.1164/RCCM.201009-1393PP. 21242594 PMC3159088

[13] Basheeruddin M. and Qausain S. , Hypoxia-Inducible Factor 1-Alpha (HIF-1α): An Essential Regulator in Cellular Metabolic Control, Cureus. (2024) 16, no. 7, 10.7759/cureus.63852, e63852. 39099978 PMC11297807

[14] Gajewski A. , Gawrysiak M. , and Szewczyk R. , et al.IL-33 Augments the Effect of Rhinovirus HRV16 on Inflammatory Activity of Human Lung Vascular Endothelium—Possible Implications for Rhinoviral Asthma Exacerbations, Allergy: European Journal of Allergy and Clinical Immunology. (2021) 76, 2282–2285, 10.1111/all.14806. 33683708

[15] Gawrysiak M. , Szewczyk R. , and Kobierecki M. , et al.Human Lung Vascular Endothelium May Limit Infection With HRV16 via IFN-β-Dependent Mechanisms, APMIS. (2024) 132, no. 2, 112–121, 10.1111/apm.13361. 37971173

[16] Green C. E. and Turner A. M. , The Role of the Endothelium in Asthma and Chronic Obstructive Pulmonary Disease (COPD), Respiratory Research. (2017) 18, no. 1, 10.1186/s12931-017-0505-1. PMC5241996 28100233

[17] Siragusa S. , Natali G. , Nogara A. M. , Trevisani M. , Lagrasta C. A. M. , and Pontis S. , The Role of Pulmonary Vascular Endothelium in Chronic Obstructive Pulmonary Disease (COPD): Does Endothelium Play a Role in the Onset and Progression of COPD?, Exploration of Medicine. (2023) 1116–1134, 10.37349/emed.2023.00199.

[18] Berger M. M. , Hesse C. , and Dehnert C. , et al.Hypoxia Impairs Systemic Endothelial Function in Individuals Prone to High-Altitude Pulmonary Edema, American Journal of Respiratory and Critical Care Medicine. (2005) 172, no. 6, 763–767, 10.1164/rccm.200504-654OC. 15947284

[19] Rossetti G. M. K. , Oliver S. J. , Sandoo A. , and Macdonald J. H. , Hypoxia-Induced Endothelial Dysfunction: Could Targeting Oxidative Stress Provide Protection?, Experimental Physiology. (2023) 108, no. 8, 1026–1028, 10.1113/EP091276. 37232510 PMC10988425

[20] Varga Z. , Flammer A. J. , and Steiger P. , et al.Endothelial Cell Infection and Endotheliitis in COVID-19, The Lancet. (2020) 395, no. 10234, 1417–1418, 10.1016/S0140-6736(20)30937-5. PMC7172722 32325026

[21] Szewczyk R. , Gawrysiak M. , Kalinowska J. , Gajewski A. , Michlewska S. , and Chałubiński M. , Human Coronavirus 229E May Infect Human Lung Vascular Endothelium and Induce Delayed Inflammatory and Antiviral Response, APMIS. (2026) 134, no. 1, 10.1111/apm.70139. 41510575

[22] Chałubiński M. , Szulc A. , and Pawełczyk M. , et al.Human Rhinovirus 16 induces Antiviral and Inflammatory Response in the Human Vascular Endothelium, APMIS. (2021) 129, no. 3, 143–151, 10.1111/apm.13103. 33230840

[23] Likońska A. , Gawrysiak M. , and Gajewski A. , et al.Human Lung Vascular Endothelium May Limit Viral Replication and Recover in Time Upon the Infection With Rhinovirus HRV16, APMIS. (2022) 130, no. 11, 678–685, 10.1111/apm.13269. 35959516

[24] Taylor C. T. and Colgan S. P. , Regulation of Immunity and Inflammation by Hypoxia in Immunological Niches, Nature Reviews Immunology. (2017) 17, no. 12, 774–785, 10.1038/nri.2017.103. PMC5799081 28972206

[25] Bartoszewski R. , Moszyńska A. , and Serocki M. , et al.Primary Endothelial Cell–specific Regulation of Hypoxia-Inducible Factor (HIF)-1 and HIF-2 and Their Target Gene Expression Profiles During Hypoxia, The FASEB Journal. (2019) 33, no. 7, 7929–7941, 10.1096/fj.201802650RR. 30917010 PMC6593883

[26] Stothers C. L. , Luan L. , Fensterheim B. A. , and Bohannon J. K. , Hypoxia-Inducible Factor-1α Regulation of Myeloid Cells, Journal of Molecular Medicine. (2018) 96, no. 12, 1293–1306, 10.1007/s00109-018-1710-1. 30386909 PMC6292431

[27] Zhao B. , Niu X. , and Huang S. , et al.TLR4 Agonist and Hypoxia Synergistically Promote the Formation of TLR4/NF-κB/HIF-1α Loop in Human Epithelial Ovarian Cancer, Analytical Cellular Pathology. (2022) 2022, 10.1155/2022/4201262, 4201262. PMC9023210 35464826

[28] Schultz K. L. W. , Troisi E. M. , Baxter V. K. , Glowinski R. , and Griffin D. E. , Interferon Regulatory Factors 3 and 7 have Distinct Roles in the Pathogenesis of Alphavirus Encephalomyelitis, Journal of General Virology. (2019) 100, no. 1, 46–62, 10.1099/jgv.0.001174. 30451651 PMC7011693

[29] Knapp S. , Yee L. J. , and Frodsham A. J. , et al.Polymorphisms in Interferon-Induced Genes and the Outcome of Hepatitis C Virus Infection: Roles of MxA, OAS-1 and PKR, Genes & Immunity. (2003) 4, no. 6, 411–419, 10.1038/sj.gene.6363984. 12944978

[30] McGettrick A. F. and O’Neill L. A. J. , The Role of HIF in Immunity and Inflammation, Cell Metabolism. (2020) 32, no. 4, 524–536, 10.1016/j.cmet.2020.08.002. 32853548

[31] Duong-Quy S. , Huynh-Anh T. , and Vo-Pham-Minh T. , et al.Management of Patients With Asthma, COPD, and OSA in Outpatient Unit: ACOSOU—Global Perspectives and Challenges, Pulmonary Therapy. (2026) 12, no. 1, 117–144, 10.1007/s41030-026-00345-2. 41557220 PMC12992749

[32] Du X. and Yang M. , Unraveling the Mechanisms of Virus-Induced Asthma Exacerbation: Epithelial Injury, Immune Dysregulation, and Novel Interventions, Chinese Medical Journal Pulmonary and Critical Care Medicine. (2025) 3, no. 3, 164–181, 10.1016/j.pccm.2025.08.003. 41114077 PMC12529573

[33] Mulpuru S. , Andrew M. K. , and Ye L. , et al.Impact of Respiratory Viral Infections on Mortality and Critical Illness Among Hospitalized Patients With Chronic Obstructive Pulmonary Disease, Influenza and Other Respiratory Viruses. (2022) 16, no. 6, 1172–1182, 10.1111/irv.13050. 36069141 PMC9530520

[34] Newton A. H. , Cardani A. , and Braciale T. J. , The Host Immune Response in Respiratory Virus Infection: Balancing Virus Clearance and Immunopathology, Seminars in Immunopathology. (2016) 38, no. 4, 471–482, 10.1007/s00281-016-0558-0. 26965109 PMC4896975

[35] King H. A. D. and Lewin S. R. , Immune Checkpoint Inhibitors in Infectious Disease, Immunological Reviews. (2024) 328, no. 1, 350–371, 10.1111/imr.13388. 39248154 PMC11659942

[36] Palacio-Castañeda V. , Velthuijs N. , Le Gac S. , and Verdurmen W. P. R. , Oxygen Control: the Often Overlooked but Essential Piece to Create Better In Vitro Systems, Lab on a Chip. (2022) 22, no. 6, 1068–1092, 10.1039/D1LC00603G. 35084420

[37] Solodushko V. , Parker J. C. , and Fouty B. , Pulmonary Microvascular Endothelial Cells Form a Tighter Monolayer When Grown in Chronic Hypoxia, American Journal of Respiratory Cell and Molecular Biology. (2008) 38, no. 4, 491–497, 10.1165/RCMB.2007-0127OC. 18048805 PMC2274951

